# Sickness Absence due to Chronic Musculoskeletal Pain: The Exploration of a Predictive Psychological Model Including Negative Moods, Subjective Health and Work Efficacy in an Adult County Population (The HUNT Study)

**DOI:** 10.5964/ejop.v14i2.1470

**Published:** 2018-06-19

**Authors:** Sven Svebak, Hallgeir Halvari

**Affiliations:** a Norwegian University of Science and Technology, Department of Mental Health, Trondheim, Norway; b University College Southeast Norway, School of Business and Social Sciences, Hønefoss, Norway; Department of Psychology, Webster University Geneva, Geneva, Switzerland

**Keywords:** dysphoria, musculoskeletal pain, sickness absence, subjective health, work efficacy

## Abstract

The relation between musculoskeletal pain and sickness absence was tested in an adult county population. Maximal explained variance in absence from work due to chronic musculoskeletal pain (sickness absence) was tested in a model in which subjective health was expected to mediate the associations between such pain and dysphoria, respectively, and work efficacy. In turn, work efficacy was expected to mediate the link between subjective health and sickness absence. All the residents in the County of Nord-Trøndelag, Norway, aged 20 and older, were invited to take part in a public health survey during 1995-97 (HUNT-2), and 66,140 (71.2%) participated. Prevalence of musculoskeletal pain, dysphoria, subjective health and work efficacy were assessed, as well as sickness absence last year due to musculoskeletal pain. The model test was performed by use of the LISREL procedure based upon data from 30,158 employees reporting chronic musculoskeletal pain last year. The measurement model fitted the data well: χ^2^ = 9075, df = 52, p < .0004, Critical N = 1041, RMSEA = 0.038, CFI = 0.99, SRMR = 0.020. The structural model fitted the data equally well, and the best prediction of sickness absence was obtained with lower back pain, upper and lower extremity pain, as well as dysphoria as the primary variables affecting subjective health that, in turn, was the convergent predictor of work efficacy that, finally, best explained the variance in sickness absence (56%). The data supported an indirect sequence of complaint-health-efficacy (CHE-model) as the best predictor of sickness absence due to musculoskeletal pain.

The pioneering report by Taylor (1968) firmly stated that the etiology of absenteeism from work involves more than strictly biomedical causes. Patient records showed that some employees with verified medical diseases subjectively reported being healthy and presented with low absenteeism, whereas many employees with no verifiable medical condition subjectively reported having poor health and scored high on absenteeism. Musculoskeletal pain is at focus in the present report as one of the major causes of absenteeism. In recent years, psychological factors have become increasingly more important in the study of causal factors, interventions and rehabilitation procedures related to work absenteeism. One example of this broadened scope of etiology is the acknowledged importance of fear of movements due to the experience that certain movements may induce pain, resulting in “fear-avoidance” that, in turn, induces passivity that causes degenerative hypotrophy in skeletal muscles and reduced load tolerance due to the related reduction of strength and endurance (Christensen et al., 2013; George, Wittmer, Fillingim, & Robinson, 2006; Grotle, Vollestad, Veierod, & Brox, 2004; Vlaeyen, Crombez, & Linton, 2016; Vlaeyen, de Jong, Geilen, Heuts, & Breukelen, 2002; Vlaeyen & Linton, 2000). Due to these developments, it has become increasingly important to avoid passivity by active and coordinated multidisciplinary support in prevention, intervention as well as rehabilitation. Accordingly, clinical procedures have been encouraged to combine medical, physiotherapeutic and psychological approaches with a focus upon overcoming fear-avoidance and other dysphoric consequences of longstanding musculoskeletal pain (Flor, Fydrich, & Turk, 1992; Sieben et al., 2005; Storrø, Moen, & Svebak, 2004).

This report is based on a county population and compared a direct effect of musculoskeletal pain on sickness absence with the modern view of indirect effects via dysphoria, subjective health and perceived work efficacy. Prevalence of age adjusted chronic musculoskeletal pain and discomfort “last year” was 44.6 percent. Among those reporting such pain, age-adjusted reduced work capacity due to this pain was reported to be 62.3 percent for men and 65.1 percent for women “last year” among a non-selected adult county population of 64,690 Norwegians (Svebak, Hagen, & Zwart, 2006). The criterion for being chronic was set to three months or more and had to be continuous over the period. The percentage of sick leave due to such pain was 25.2 for men and 27.7 for women, which meant that almost 75% of people with enduring musculoskeletal pain reported no sickness absence “last year” due to this problem. Neck, shoulder and lower back pain were the most prevalent, with corresponding percentages of 13.5, 15.7 and 13.4 for men and 18.7, 20,2 and 16.9 for women. In this adult county population, there was an effect upon sick leave of number of body areas with chronic pain. Only one area, such as the lower back, resulted in 25.2% of the males and 27.7% of the females reporting sick leave, whereas those reporting pain in seven or more body areas reported around 50% of sick leave last year. Interestingly, prevalence of chronic headache (migraine and non-migrainous) was more than four times higher (OR = 4.6; CI 4.0-5.3) in those adults who reported musculoskeletal symptoms than in those without in this county cohort (Hagen, Einarsen, Zwart, Svebak, & Bowim, 2002), thus indicating a more complex role for pain in sickness absence due to headache than due to pain restricted to the skeletal muscles, and neck pain was more strongly associated with headache than pain from other muscle sites.

It has long been assumed that psychological characteristics can buffer as well as mediate pain sensitivity (Melzack, 1999; Melzack & Wall, 1965). A number of brain neurotransmitters are involved in pain perception. They also are involved in dysphoria. Such transmitters include serotonin and, therefore, offer treatment by reuptake inhibitors in depression that often is the consequence of chronic musculoskeletal pain (Basbaum & Jessell, 2000). Thus, undiagnosed psychiatric disorders are frequent in chronic musculoskeletal pain, as seen in scores on the Beck Depression Inventory (Olaya-Contreras, Persson, & Styf, 2010). Moreover, the endogenous opioids have been explored over the last twenty years (Bruehl, Carlson, Wilson, & Norton, 1996). All implicated neural mechanisms involved in pain perception influence mood, attention and cognition, as proposed in the dynamics of the fear-avoidance model where negative affect and harm representation maintains nociception, whereas positive affect and optimism mediate recovery (Vlaeyen, Crombez, & Linton, 2016). These recent insights favor an indirect, rather than a direct, effect of musculoskeletal pain on sickness absence.

It is assumed that coping resources are challenged when individuals are faced with the stressor of pain in everyday life and, consequently, are reflected in perceived self-efficacy (Bandura, 1997) that, again, is influenced by moods (Bower, 1981; Eich, 1995) including anxiety and depression (Alloy, Clements, & Koenig, 1993). In this way, negative moods challenge mental resources for pain control (Bandura, Cioffi, Taylor, & Brouillard, 1988) that also relate to personality dispositions such as neuroticism and the sense of humor (Svebak et al., 2000). Increase of perceived self-efficacy has long ago been proven to influence sickness absenteeism. Pioneering studies of selected samples involved programs for the development of self-regulation of own motivation to handle personal and social problems, including the development of behavioral skills, and resulted in significant reduction of sickness absence (Frayne & Latham, 1987; Latham & Frayne, 1989). The present study explored the predictive power of a Complaint-Health-Efficacy (CHE) model in explaining the absence from work (sickness absence) due to chronic musculoskeletal pain. Based upon previous research, pain in the neck, shoulders and lower back as well as mental complaints (dysphoria) were seen as primary sources of risk, with pain in the upper and lower extremities as additional risks of reduced subjective health that, again, influence the subjective perception of work efficacy that, finally, is a major cause of sickness absence.

## Method

### Study Population

The present study is based on data from one of the largest population health surveys in the world. Between 1995 and 1997, all inhabitants above 19 years in the County of Nord-Trøndelag in Norway were invited to participate in the Nord-Trøndelag Health Study (HUNT-2) described in detail elsewhere (Holmen et al., 2003). Out of 92,936 invited individuals, 66,140 (71.2%) participated, with more women than men in all age groups under 70. Participation was age dependent, with highest participation in the age group 60-69 for both sexes (men: 84.3%; women: 87.0%) and gradually lower participation rates in the younger and older groups. The county population is fairly representative of Norway as a whole, except that there is no large city, and the average educational and income levels are somewhat lower than the nation average. The population is stable and ethnically homogeneous with only a minor percentage (3%) of Lapps and people of non-Caucasian origin.

A non-participation study was performed in a 2.5% random sample of non-attendants. The main reasons for not attending in the age group 20-44 were lack of time or having moved out of the county. In the age group 70+ many reported to have regular follow-up by a doctor or hospital and, therefore, did not need to attend the health survey (Holmen et al., 2003).

### Survey Measures and Procedure

For all eligible inhabitants, a questionnaire (Q1) was enclosed along with an invitation to a clinical examination. When attending to the screening site, the participants received a second questionnaire (Q2) to complete and return by mail. Both questionnaires included an extensive set of variables addressing health issues, and both Q1 and Q2 included questions about musculoskeletal pain adopted from the Standardized Nordic Questionnaire. In the present study, however, data only from the Q1 are at the basis for prediction of sickness absence. The Standardized Nordic Questionnaire has previously been validated and found to give reliable estimates for lower back, upper extremities as well as neck pain, in particular for symptoms during the past year (Franzblau, Salerno, Armstrong, & Werner, 1997; Kuorinka et al., 1987; Palmer, Smith, Kellingray, & Cooper, 1999). To our knowledge, information about pain in other parts of the body has not been validated.

In Q1 participants who responded “yes” to the question “Have you during the last year continuously for at least three months suffered from pain or stiffness in muscles and joints?” were defined as having chronic musculoskeletal pain (*N* = 30,158). They were asked to indicate locations by “yes” (2) or “no” (1) responses to the following areas of the body: Neck, shoulders, elbows, wrists/hands, chest/abdomen, upper back, lower back, hips, knees, and/or ankles/feet. They also responded to a question on if pain had reduced their work capacity last year (including home work), with four response alternatives: No/hardly noticeable (3) - To some degree (2) - To a great degree (1), or Do not know. This variable was labeled “Work efficacy.” They were also asked if pain had forced them on sick leave by responding: “Yes” (2) or “No” (1); note that N may be less than 30,158 in some analyses due to lack of relevance of certain pain area variables for any particular individual or due to missing data.

One item in Q1 assessed subjective health by asking: “How is your current health?” Answers were given according to a four-step format: Poor - Not too good - Fair - Very good, weighted by 1 to 4, respectively. A four-step format also assessed prevalence of dysphoric moods over “the last couple of weeks” by two questions: “Have you felt nervous and tense?” and “Have you suffered from anxiety?” (No - To some degree - Quite a bit - Very much, weighted by 1 to 4, respectively, for both items). Memory for moods appears to be fair over the past few weeks (Bower, 1981).

### Data Scoring and Statistical Analyses

Scores on chronic musculoskeletal pain (sum of scores on two items), dysphoric moods (sum of scores on two items), and single-item variables of subjective health and work efficacy were all included as predictors of sickness absence. Analyses were performed by the LISREL statistical package (Bollen, 1989) and explored the sequence and relationships among these predictors for best explanatory power in the latter (dependent) variable.

## Results

### Descriptive Statistics

It will be seen in Table 1 that skewness was fair in all variables except for dysphoric moods where most of the population reported relative absence of tension and anxiety over the weeks preceding their responding to the survey (see note). In variables with two items comprising the model index, Cronbach´s alpha supported homogeneity of the index.

**Table 1:** Means (M), Standard Deviations (SD) and Zero Order Correlations Among Variables

Variable	*M*	*SD*	Skewness	1	2	3	4	5	6	7	8	9	10
1. Neck/shoulder	1.78	0.41	-1.26	.81									
2. Lower back	1.63	0.41	-0.49	.27	.78								
3. Dysphoria	1.36	0.60	2.23	.09	.09	.82							
4. Upper extremities	1.42	0.43	0.36	.30	.35	.11	.72						
5. Lower extremities	1.50	0.44	0.17	.20	.48	.12	.54	.72					
6. Subjective health	2.57	0.65	-0.17	-.15	-.32	-.26	-.18	-.36	-				
7. Work efficacy	2.02	0.74	-0.01	-.11	-.33	-.17	-.30	-.31	.46	-			
8. Absenteeism	1.27	0.44	1.07	.08	.18	.06	.18	.13	-.23	-.45	-		
9. Sex^a^	1.57	0.50	-.01	.11	.19	.08	.15	.16	-.05	-.05	.03	-	
10. Age	53.21	16.06	0.37	.06	.16	.01	.10	.26	-.31	-.20	-.09	.02	**-**

*Note.* Reliability coefficients for variables 1-5 are given in the diagonal as Cronbach´s alpha.

^a^1 = males; 2 = females.

### Bivariate Relations

Table 1 also presents a correlation matrix for all variables included in the study. Physical (pain) and mental (dysphoria) complaints were negatively correlated with subjective health that, in turn, was positively correlated with work efficacy. The complaint and subjective health variables were positively and negatively associated with sickness absence, respectively. Work efficacy was most strongly correlated with sickness absence.

### Structural Equation Models Tested in LISREL

**Figure 1:**
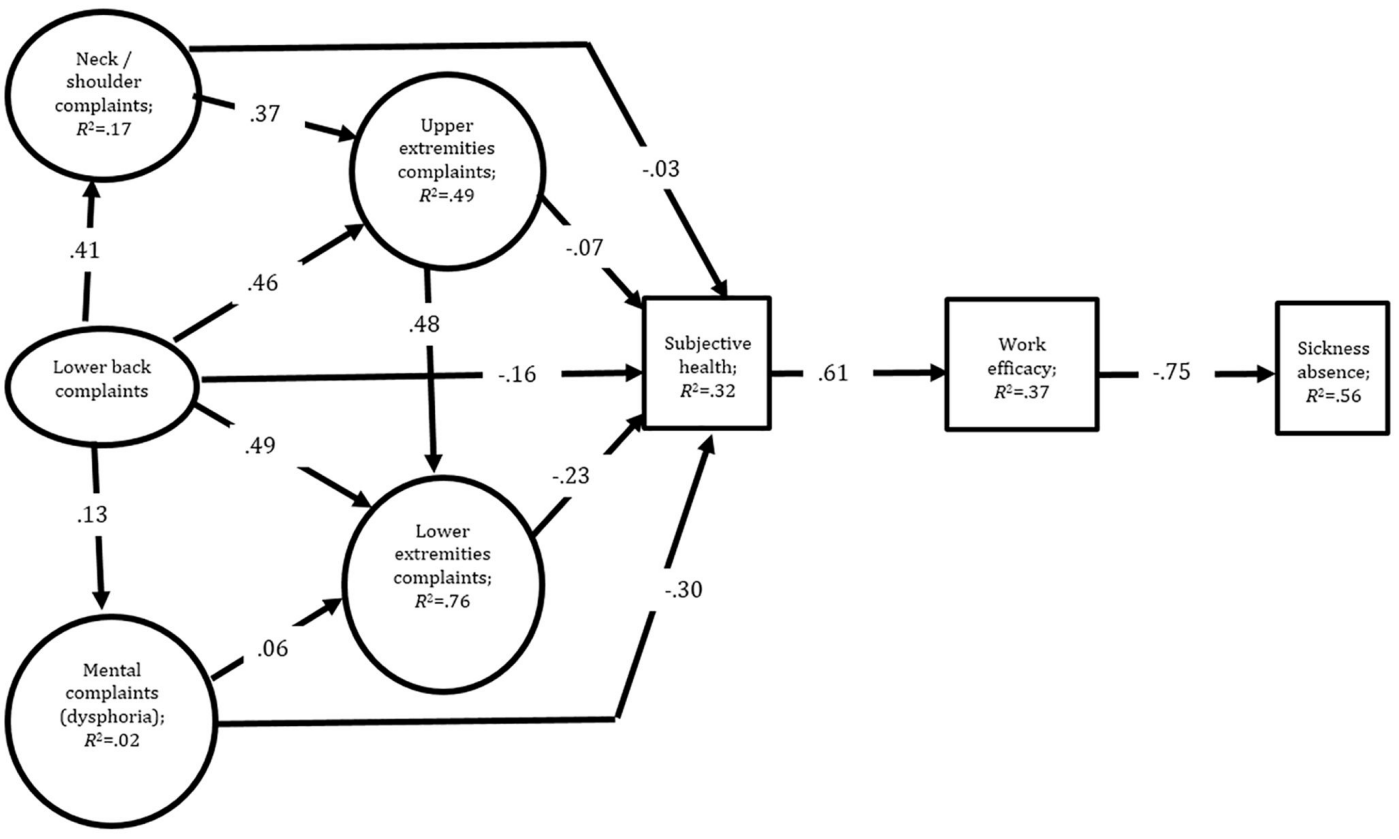
Standardized parameter estimates are given for the structural model. All paths are significant at or beyond *p* < .001. Note that the explained variance in each given path is calculated as the squared estimate. Factor loadings for the indicators of latent variables (ovals) are given in the method section.

According to Anderson and Gerbing (1988), the measurement model at large should first be tested for overall acceptable fit before testing the power of the structural model that is presented in Figure 1. This requirement was met by a confirmatory factor analysis (maximum likelihood) on the specified relations and lack of significant relations between the latent variables. Model fit indices were the chi-square likelihood ratio (χ^2^) and its *p*-value, the Root Mean Square Error of Approximation (RMSEA), the Comparative Fit Index (CFI) and the Standardized Root-Mean-Square Residual (SRMR). These different indices are recommended in order to evaluate model fit in covariance structure analyses (Bollen, 1989; Byrne, 2001; Hu & Bentler, 1999; Kline, 1998). A good fit should have a value close to or lower than .06 for the RMSEA, a value close to or lower than .08 for the SRMR, combined with a value close to or higher than .95 for the CFI, and the *p*-value of the chi-square should be .05 or above. However, when the sample is large, as in the present study, the *p*-value of the chi-square is a poor fit estimate. Thus, with large samples it is suggested that the critical-*N* statistics should exceed 200 for accepting the fit of a model for the chi-square test (Hoelter, 1983). Hu and Bentler (1999) compared all fit indices and found that the SMSR is most sensitive to misspecification in simple as well as complex models and less sensitive to sample size and violations of distributional assumptions.

The measurement model did fit the data well (χ^2^ = 9075.82, *df* = 52, *p* < .0004, critical *N* = 1041, RMSEA = 0.038, CFI = 0.99, IFI = 0.99, SRMR = 0.020). This final model included all indicators (items) for the latent measures (variables) that are described in the method section. Item reliabilities indicated by squared factor loadings were for neck / shoulder .83 and .88, for lower extremities pain .75 and .77, for upper extremities pain .70 and .78, for lower back pain .64 and .89, and for the index of dysphoria .82 and .98. For the observed single item variables, the error variance was set to 15% (reliability = .85, which is expected for variables like subjective health and work efficacy) of the squared standard deviation for each variable. When using LISREL this is recommended because the error variance for single-item construct indicators must be assumed (Anderson & Gerbing, 1988, p. 415; Hayduk, 1987, pp. 119-123).

### The Complaint-Health-Efficacy Process Model of Sickness Absence

Hypotheses were tested by structural equation modeling (LISREL) for the relations among variables in the process model that appears in Figure 1. The model fitted the data well (χ^2^ = 13574.84, *df* = 66, *p* < .0004, critical *N* = 666, RMSEA = 0.47, CFI = 0.98, IFI = 0.98, SRMR = 0.036). The standardized parameter estimates are given in Figure 1.

### Tests of Indirect Associations

The indirect links emerging in Figure 1 were tested in LISREL (see Table 2). The results indicated that all of them were significant. Estimation of indirect effects was performed by the multiplication of the unstandardized parameter estimates in indirect associations. A sample illustration of the indirect effect of subjective health on sickness absence is obtained by multiplying the unstandardized parameter estimate, linking subjective health to work efficacy, by the unstandardized parameter estimate linking work efficacy to sickness absence (Diamantopoulos & Siguaw, 2000).

**Table 2:** LISREL Tests of Indirect Associations Emerging in Figure 1.

Independent variable		Mediator		Dependent variable	Indirect effect	*SE*	*Z*	95% *CI*	
								Lower	Upper
1. Lower back pain	➔	Neck/Shoulder pain	➔	Upper extremities pain	0.33	0.004	102.4***	0.322	0.338
2. Lower back pain	➔	Upper extremities pain	➔	Lower extremities pain	0.77	0.005	168.0***	0.760	0.780
3. Lower back pain	➔	Mental complaints	➔	Lower extremities pain	0.04	0.005	26.4***	0.032	0.052
4. Lower back pain	➔	Lower extremities pain	➔	Subjective health	-0.02	0.040	-104.2***	0.248	0.232
5. Lower back pain	➔	Mental complaints	➔	Subjective health	-0.05	0.005	-10.9***	0.070	0.060
6. Lower back pain	➔	Subjective health	➔	Work efficacy	-0.22	0.004	-104.6***	0.228	0.212
7. Neck/shoulder pain	➔	Upper extremities pain	➔	Lower extremities pain	0.48	0.005	126.8***	0.470	0.490
8. Neck/shoulder pain	➔	Upper extremities pain	➔	Subjective health	-0.12	0.010	-69.7***	0.140	0.100
9. Neck/shoulder pain	➔	Subjective health	➔	Work efficacy	-0.10	0.010	-55.5***	0.120	0.080
10. Mental complaints	➔	Lower extremities pain	➔	Subjective health	-0.06	0.004	-49.3***	0.068	0.052
11. Mental complaints	➔	Subjective health	➔	Work efficacy	-0.18	0.004	-92.2***	0.188	0.172
12. Upper extremities pain	➔	Lower extremities pain	➔	Subjective health	-0.22	0.005	-100.8***	0.230	0.210
13. Upper extremities pain	➔	Subjective health	➔	Work efficacy	-0.21	0.010	-102.6***	0.230	0.190
14. Lower extremities pain	➔	Subjective health	➔	Work efficacy	-0.20	0.004	-100.1***	0.208	0.192
15. Subjective health	➔	Work efficacy	➔	Sickness absence	-0.76	0.011	-132.7***	0.782	0.758

*Note.* CI = Confidence interval.

***p < .001.

## Discussion

The findings clearly supported the indirect effects of musculoskeletal pain on work efficacy, and perceived work efficacy was the best mediator between subjective health and sickness absence.

The present data were based on self-report. A recent meta-analysis (Johns & Miraglia, 2015) examined the test-retest reliability, convergent validity and accuracy of self-reported absence. It was concluded that test-retest reliability was adequate and that, compared with organizational data, employees tended to underreport their absenteeism although the validity is fairly good. Interestingly, accuracy was boosted when sickness absence was tested, compared with absence for any reason. A total of thirty-four studies were included in their analyses.

The zero order correlations reported in Table 1 were all in line with theoretical expectations and empirical findings (see introduction). From this perspective, the present findings from an adult county population corroborated research on psychological factors that influence the relation between subjective health, perceived work efficacy and sickness absence. However, the present complaint-health-efficacy model (CHE-model) was more powerful in predicting sickness absence than we expected. It is rare in empirical research to be able to explain more than half (.75^2^ = 56%) of the variance in the target variable by a model applied to data derived from an adult population (see Figure 1). Therefore, the results may be seen as a strong support to the powerful role of psychological variables as mediators in the explanation of sickness absence due to musculoskeletal pain.

Only 9% of the variance in subjective health was uniquely explained by dysphoria. This variable reflects a much broader approach to negative moods than is assessed by fear-avoidance that explicitly orients to fear of movement-induced pain. Taken together, dysphoria and musculoskeletal pain explained only 32% of the variance in subjective health. The present findings clearly illustrate the importance of searching for other sources of sickness absence attributed to musculoskeletal pain than the fear of pain itself. For example, Vlaeyen, Crombez, and Linton (2016) argued that pain is not always associated with avoidance. The context often involves competing goals where the value of another outweighs the value of avoiding pain. This point represents a strong basis for rehabilitation to focus, not on negative affect, pain and risk of harm, but on positive affect, coping and optimism. In the clinical setting, the importance of this shift from a negative spiral to a positive one was described by Svebak (2007).

Self-efficacy theory defines three main sources of perceived efficacy: Personal and collective efficacy as well as efficacy to exercise control behaviorally (Bandura, 1997). The collective and behavioral sources of efficacy were not included in the HUNT population study and, therefore, may represent some of the unexplained variance in the CHE-model. Resent publications have pointed out the importance of personality and job characteristics for sickness absence. Thus, Ree et al. (2014), from the perspective of response outcome expectancies, investigated the effects of socioeconomic status and physical and psychosocial workload upon subjective health. They concluded that the level of perceived coping (helplessness) is a significant mediator in the relationship of physical workload and subjective health. The present dysphoria measure reflected poor coping with more than load at work, but addressed no personality differences, whereas Hystad, Eid, and Brevik (2011) addressed the effects of psychological hardiness, job demands and job control on sickness absence among 7,239 employees. They concluded, after controlling for age, sex and baseline absence, that baseline hardiness predicted sickness absence. A complex interaction was observed due to more sickness absence among those scoring low on hardiness, when job demands were high among those who also reported high job control. Such interactions were not tested in the present approach and may further contribute to the unexplained variance in subjective health and in work efficacy.

Results from other studies of work efficacy have supported its predictive role in sickness absence. One of these studies followed a Danish cohort of 5,357 employees including 106 employees with long-term sickness absence (Labriola et al., 2007). They concluded from their prospective study that self-efficacy was lower among employees with sickness absence than in the general working population, but no significant association was reported for self-efficacy and a later onset of sickness absence or with return-to-work. Interestingly, these authors suggested that reduced self-efficacy is the consequence of sickness absence as much as a precursor. Another study of the relationship between self-efficacy and sickness absence followed 233 Swedish employees with long-term sickness absence due to musculoskeletal pain (Busch, Göransson, & Melin, 2007). The results stated that those with negative recovery beliefs, a low sense of mastery, perceived high mental demands at work and prior experiences of long-term sickness absence, had increased probabilities of being in need of sickness benefits at work. Long-term sickness strongly increased a sense of helplessness, reduction in self-efficacy and a reduced probability of returning to work. Also in the present multivariate correlational study, perceived work efficacy emerged as a powerful predictor of sickness absence and, therefore, calls upon interventions with a focus on the improvement of perceived work-related efficacy.

People with a high sense of general self-efficacy feel frustrated by limited opportunities at work and by organizational constraints that reduce collective work efficacy (Jex & Gudanowski, 1992). However, the present study dealt with personal efficacy to fulfill job demands. Low such efficacy typically reflects anxiety and other underlying dysphoric moods triggered by, if not also causing, musculoskeletal pain. The seemingly strong impact of these variables on subjective health and work efficacy, calls upon every effort to provide personal work environments that facilitate a sense of accomplishment. Obviously, the perception of work efficacy is the consequence of personal *as well as* organizational efforts with a broad approach including social support at work (Sommer, Svendsen, & Frost, 2016) in helping employees to develop a sense of efficacy across the personal, social as well as behavioral domains. In such efforts, one should keep in mind that the effects may be due to complex processes. Thus, de Jonge, Reuvers, Houtman, Bongers, and Kompier (2000) reported both linear and nonlinear relations between psychosocial job characteristics, subjective outcomes and sickness absence in a study of musculoskeletal pain. In their three-year prospective study, baseline data supported a linear additive model for health complaints and sickness absence, whereas a curvilinear model was superior for emotional exhaustion and depression. Their initial sample of 2,064 employees was drawn from various sectors including industry and services in the Netherlands. Clearly, there are complex processes addressed in the efforts to reduce sickness absence, and they are not all explicitly defined in the CHE-model described in Figure 1, although most of the complexity appeared to be located in the transitions between complaints and subjective health as well as between subjective health and self-efficacy. These unaddressed complexities notwithstanding, the latter variable explained a high level of variance in sickness absence.

The present results and those reported by Busch et al. (2007), Labriola et al. (2007), de Jonge et al. (2000) and others, taken together, suggest a dynamic cascade of events where reduced work efficacy, for reasons that may or may not relate to work, strongly increase the risk of sickness absence that may further induce a perception of reduced self-efficacy that, again, increases the risk of long-term sickness absence. Efforts to maintain and increase self-efficacy are clearly supported in the present as well as other reported findings.

A final note on the qualities of the present study would have to include the unusual size and the relatively unselected nature of the population that was addressed. The high number of subjects offered powerful statistics in the multivariate model testing. It also should be underlined that the present methodological approach is basically correlational and, therefore, calls upon caution in conclusions about causal relationships.

In conclusion, the CHE-model supported a role for mediating psychological variables in the relation between musculoskeletal pain and sickness absence. This role has been increasingly supported in research over the last fifty years. Never before has this mediating role for psychological variables been statistically supported in data from a relatively unselected adult county population.
